# *Moringa oleifera* Leaf Petroleum Ether Extract Inhibits Lipogenesis by Activating the AMPK Signaling Pathway

**DOI:** 10.3389/fphar.2018.01447

**Published:** 2018-12-18

**Authors:** Jing Xie, Yan Wang, Wei-Wei Jiang, Xuan-Fei Luo, Tian-Yi Dai, Lei Peng, Shuang Song, Ling-Fei Li, Liang Tao, Chong-Ying Shi, Ruo-Shi Hao, Rong Xiao, Yang Tian, Jun Sheng

**Affiliations:** ^1^ Yunnan Provincial Key Laboratory of Biological Big Data, Yunnan Agricultural University, Kunming, China; ^2^ College of Biological Big Data, Yunnan Agricultural University, Kunming, China; ^3^ College of Food Science and Technology, Yunnan Agricultural University, Kunming, China; ^4^ College of Science, Yunnan Agricultural University, Kunming, China; ^5^ Research Institute of Plateau Characteristic Agricultural Industry, Kunming, China; ^6^ Key Laboratory of Pu-er Tea Science, Ministry of Education, Yunnan Agricultural University, Kunming, China

**Keywords:** *Moringa oleifera* leaf petroleum ether extract, 3T3-L1 adipocytes, adipogenesis, lipolysis, AMPK, antiobesity

## Abstract

In recent years, obesity has become a key factor affecting human health. *Moringa oleifera* Lam. is a perennial tropical deciduous tree, which is widely used in human medicine due to its nutritional and unique medicinal value. It has a cholesterol-lowering effect, but its mechanism of action is unclear. In this study, we elucidated the inhibitory effect of *M. oleifera* leaf petroleum ether extract (MOPEE) on lipid accumulation by *in vitro* and *in vivo* experiments, and we described its mechanism of action. MOPEE suppressed adipogenesis in 3T3-L1 adipocytes in a dose-dependent manner and had no effect on cell viability at doses up to 400 μg/ml. Furthermore, MOPEE (400 μg/ml) significantly downregulated the expression of adipogenesis-associated proteins [peroxisome proliferator-activated receptor γ (PPARγ), CCAAT/enhancer-binding proteins α and β (C/EBPα and C/EBPβ), and fatty acid synthase (FAS)] and upregulated the expression of a lipolysis-associated protein [hormone-sensitive lipase (HSL)] in 3T3-L1 adipocytes. Additionally, MOPEE (400 μg/ml) significantly increased the degree of phosphorylation of AMP-activated protein kinase α (AMPKα) and acetyl-CoA carboxylase (ACC). An AMPK inhibitor reversed the MOPEE-induced activation of AMPKα and ACC in 3T3-L1 adipocytes. Animal experiments showed that, in high-fat diet (HFD) mice, MOPEE [0.5 g/kg body weight (BW)] effectively decreased BW; relative epididymal, perirenal, and mesenteric fat weight and fat tissue size; and hepatic fat accumulation. Furthermore, MOPEE markedly reduced the serum levels of total cholesterol (TC), low-density lipoprotein cholesterol (LDL-C), and aspartate aminotransferase (AST). Moreover, MOPEE significantly downregulated the expression of adipogenesis-associated proteins (PPARγ and FAS) and upregulated the expression of a lipolysis-associated protein [adipose triglyceride lipase (ATGL)] in HFD mice hepatic and epididymal fat tissue. Additionally, MOPEE markedly increased the degree of phosphorylation of AMPKα and ACC in HFD mice hepatic and epididymal fat tissue. Following ultrahigh-performance liquid chromatography quadrupole time-of-flight tandem mass spectrometry (UPLC-QTOF-MS/MS) analysis, three phytocompounds (isoquercitrin, chrysin-7-glucoside, and quercitrin) were identified as compounds with relatively high levels in MOPEE. Among them, quercitrin showed excellent fat accumulation inhibitory activity, and the three compounds had synergistic effects in inhibiting adipogenesis. Taken together, MOPEE inhibits fat accumulation by inhibiting the adipogenesis and promoting the lipolysis, and this process is related to AMPK activation.

## Introduction

Metabolic syndrome is a clustering of risk factors, such as central obesity, insulin resistance, dyslipidemia, and hypertension, which together culminate in increased risk of type 2 diabetes mellitus and cardiovascular disease (O’Neill and O’Driscoll, 2015). Truncal obesity plays an exceptionally critical role among all the metabolic traits of the metabolic syndrome, and the prevalence of metabolic syndrome has steadily increased with the growing epidemic of obesity (Martin et al., 2015). The occurrence of obesity is closely associated with genetic and environmental factors; environmental factors mainly include eating habits and lifestyle (Lai et al., 2015). Obesity is characterized by pathologic growth of adipose tissues to accommodate excess energy intake by increasing the volume and number of adipocytes in the body. The proliferation and differentiation of preadipocytes play an extremely important role in this process. Thus, inhibiting the preadipocyte proliferation and differentiation (adipogenesis) is one of the methods to prevent and treat obesity (Figarola and Rahbar, 2013).

Preadipocytes originate from mesenchymal stem cells in adipose tissue. In the process of differentiation of preadipocytes into mature adipocytes, many transcription factors need to be continuously activated, including CCAAT/enhancer-binding proteins (C/EBPs), peroxisome proliferator-activated receptor (PPAR), and sterol regulatory element-binding protein 1 (SREBP1) (Lai et al., 2015; Sharma et al., 2015; Liou et al., 2017). The characteristic of adipogenesis is the accumulation of triglyceride (TG), during which the expression of various enzymes increases, accompanied by an increase in lipogenesis and/or a decrease in lipolysis. Fatty acid synthase (FAS) is a key enzyme in fatty acid synthesis in adipocytes. It is mainly associated with the synthesis of endogenous fat in cells and is involved in the differentiation of adipocytes. It is highly expressed in the later stages of the process in which fat cells differentiate from fibroblasts to mature adipocytes, and it is closely related to the formation of lipid droplets in adipocytes. Adipose triglyceride lipase (ATGL) and hormone-sensitive lipase (HSL) are the main hydrolyzing enzymes that hydrolyze TG and diglycerides, accounting for 95% of lipolytic enzyme activity in white adipose tissue (Nielsen et al., 2014).

AMP-activated protein kinase (AMPK) is a key regulator of cellular glucose and lipid metabolism, and its activation enhances insulin sensitivity in various tissues, promotes energy generation (glucose transport and fatty acid oxidation), inhibits energy expenditure (lipid synthesis, protein synthesis, and gluconeogenesis), inhibits fatty acid release in adipocytes, promotes fatty acid degradation, and thus plays a role in the therapeutic effect on metabolic syndrome, diabetes, etc. (Daval et al., 2006). Acetyl-CoA carboxylase (ACC) is a downstream molecule of AMPK that regulates the synthesis of fatty acids, catalyzes the production of malonyl-CoA from acetyl-CoA, and catalyzes the synthesis of long-chain fatty acid precursors (Mottillo et al., 2017). Phosphorylation of AMPK promotes phosphorylation of ACC, resulting in inactivation of ACC, thereby reducing the free fatty acid production. In addition, AMPK inhibits the synthesis of fatty acids and cholesterol by inhibiting the transcription of SREBP1c and FAS (Bullon et al., 2016). Therefore, regulating AMPK activation and the expression of its downstream target proteins have become new approaches for the treatment of obesity.

Currently, many kinds of agents are used to prevent or treat obesity, but some have side effects, including gastrointestinal discomfort, flatulence, diarrhea, insomnia, and headache (Lee et al., 2015). Therefore, it is important to study the antiobesity effects of plants and their natural products. *Moringa oleifera* Lam. (of the Moringaceae family) is a type of medicinal Indian herb that is widely known in tropical and subtropical countries (Abdull Razis et al., 2014). Currently, many studies have revealed the health effects of *M. oleifera*, including cholesterol-lowering, antidiabetic, antihypertensive, antitumor, anti-inflammatory, antioxidant, antipyretic, antiepileptic, antiulcer, antispasmodic, diuretic, hepatoprotective, antibacterial, and antifungal activity (Ndong et al., 2007; Sangkitikomol et al., 2014; Yassa and Tohamy, 2014). Simultaneously, many studies have reported the antiobesity and antihyperlipidemic effects of *M. oleifera* (Ghasi et al., 2000; Ndong et al., 2007; Sangkitikomol et al., 2014; Helmy et al., 2017). However, the lipogenesis inhibitory effect of *M. oleifera* leaf petroleum ether extract (MOPEE) and its mechanism of action *in vitro* and *in vivo* remain inconclusive. In this study, we investigated the inhibitory effects of MOPEE on lipogenesis by *in vitro* and *in vivo* experiments, and we explored the potential mechanisms underlying these effects.

## Materials and Methods

### Preparation of the *M. oleifera* Leaf Extract and Ultrahigh-Performance Liquid Chromatography Quadrupole Time-of-Flight Tandem Mass Spectrometry (UPLC-QTOF-MS/MS) Analysis

*M. oleifera* leaves (10 kg, Yunnan God Bless Technology Development Co., Ltd., Dehong, China) were air-dried and pulverized. The dried powder was extracted three times with 75% acetone under soaked at room temperature. The combined extract was removed at 50°C under vacuum to afford a viscous residue. The extract was diluted with water and then partitioned three times with petroleum ether. The petroleum ether-soluble materials were concentrated using reduced pressure to recover the petroleum ether, and 30 g (0.3%) *M. oleifera* residue was obtained [*M. oleifera* leaf petroleum ether extract (MOPEE)]. Water-soluble materials were extracted three times with chloroform. The chloroform-soluble materials were concentrated using reduced pressure to recover the chloroform, and 200 g *M. oleifera* residue was obtained [*M. oleifera* leaf chloroform extract (MOCE)], with a yield from the dried *M. oleifera* leaves of approximately 2.0%. Subsequently, water-soluble materials were extracted three times with n-butyl alcohol. The n-butyl alcohol was recovered using reduced pressure to obtain 200 g (2%) *M. oleifera* residue [*M. oleifera* leaf n-butyl alcohol extract (MONAE)]. The remaining water-soluble materials were concentrated to obtain 100 g (1%) *M. oleifera* residue [*M. oleifera* leaf water extract (MOWE)].

The composition of the MOPEE was analyzed using an Agilent 1290 UPLC-QTOF-MS/MS system (Agilent, CA, USA). The liquid-phase conditions were as follows: diode array detector (DAD); Zorbax Eclipse Plus C18 column (2.1 × 100 mm, 1.8 μm; Agilent); elution solvents: 0.1% formic acid water 1) and acetonitrile 2); flow rate: 0.3 ml/min; column temperature: 30°C; injection volume: 1 μl; gradient elution: 0–15 min, 10–25% B; 15–16 min, 25–35% B; 16–38 min, 35–55% B; 38–42 min, 55% B. The mass spectrometry conditions were as follows: electrospray ionization (ESI) negative ion mode; ion range: m/z 100–1,700; collision gas: nitrogen; drying gas flow rate: 10.0 L/min; drying gas temperature: 300°C; atomization gas pressure: 30 psi; capillary voltage: 3.5 kV; collision voltage: 175 V; MS2 collision energy of the parent ion: 10–50 V.

### 3T3-L1 Preadipocyte Culture and Differentiation

3T3-L1 preadipocytes (Shanghai Cell Institute Country Cell Bank, Shanghai, China) were grown in ordinary medium [Dulbecco’s Modified Eagle Medium/Nutrient Mixture F-12 (DMEM/F-12) supplemented with 10% fetal bovine serum and 1% penicillin/streptomycin (1,000 units of penicillin/ml and 1,000 μg streptomycin/ml)] at 37°C in 5% CO_2_ atmosphere. The differentiation of 3T3-L1 preadipocytes was accomplished by the following method. Briefly, 4 ml of the 3T3-L1 preadipocyte suspension (5 × 10^4^ cells/ml) was seeded onto 50 mm dishes. After 48 h, the ordinary medium was replaced with differentiation medium (ordinary medium supplemented with 5 μg/ml insulin, 500 μM 3-isobutyl-1-methylxanthine, 1 μM dexamethasone, and 2 μM rosiglitazone). After 8 days of culture, the differentiation medium was replaced with insulin medium (ordinary medium supplemented with 5 μg/ml insulin) and cultured for another 4 days.

### Cell Viability Assay

After 3T3-L1 preadipocytes were differentiated into adipocytes as described above, the effect of MOPEE on adipocyte viability was evaluated using a cell counter (Countess® Automated Cell Counter, Invitrogen, CA, USA). In brief, differentiated adipocytes were treated with MOPEE, MOCE, MONAE, or MOWE (0, 25, 50, 100, 200, 400 μg/ml) for 24 h, respectively. After digestion with trypsin, cell viability was measured using the cell counter. The results were normalized to the control (untreated cells).

The effect of isoquercitrin, chrysin-7-glucoside, quercitrin, and combinations of these compounds on cell viability was determined by using a 3-(4,5-dimethylthiazol-2-yl)-2,5-diphenyltetrazolium bromide (MTT) assay. 3T3-L1 adipocytes were seeded in 96-well plates at a density of 2 × 10^4^ cells/well. They were cultured for 24 h with isoquercitrin (36.4 and 72.8 μg/ml), chrysin-7-glucoside (14 and 28 μg/ml), quercitrin (9.2 and 18.4 μg/ml), combination 1 (36.4 μg/ml isoquercitrin + 14 μg/ml glucoside + 9.2 μg/ml quercitrin), and combination 2 (72.8 μg/ml isoquercitrin + 28 μg/ml glucoside + 18.4 μg/ml quercitrin). MTT (5 mg/ml) solution was added to each well and incubated for 4 h at 37°C. The formazan formed was measured at 492 nm using a microplate reader (Molecular Devices Co., CA, USA).

### Oil Red O Staining

3T3-L1 adipocytes were treated with MOPEE (0, 25, 50, 100, 200, 400 μg/ml) for 24 h. Subsequently, the cells were washed twice with phosphate-buffered saline (PBS) and fixed with 4% paraformaldehyde for 30 min at room temperature. Excess 4% paraformaldehyde was removed, the cells were washed twice with PBS, and staining was performed with 60% oil red O dye (Sigma, St. Louis, MO, USA) for 10 min. Excess oil red O was removed, and the cells were then washed twice with PBS. The lipid droplets within the cells were visualized and photographed by inverted microscopy (Olympus, Tokyo, Japan).

Fresh hepatic tissues were embedded in tissue-freezing medium (Cell Path Ltd., Newtown, UK) and stored at −80°C. Subsequently, 8-μm frozen sections were fixed with 10% formaldehyde solution for 1 h at 4°C. Excess formaldehyde solution was removed, and the sections were washed three times with PBS. Staining was performed with oil red O dye for 15 min in the dark, and the sections were then rinsed with distilled water, stained with hematoxylin dye for 16 s, and rinsed again with distilled water for 5–10 min. The sections were observed and photographed by inverted microscopy (Olympus).

### TG Assay

3T3-L1 adipocytes were treated with MOPEE, MOCE, MONAE, or MOWE (0, 25, 50, 100, 200, 400 μg/ml), isoquercitrin (36.4 and 72.8 μg/ml), chrysin-7-glucoside (14 and 28 μg/ml), quercitrin (9.2 and 18.4 μg/ml), combination 1 (36.4 μg/ml isoquercitrin + 14 μg/ml glucoside + 9.2 μg/ml quercitrin), and combination 2 (72.8 μg/ml isoquercitrin + 28 μg/ml glucoside + 18.4 μg/ml quercitrin) for 24 h. The adipocytes were then collected, and 2% TritonX-100 was added to lyse the cells for 40 min. Quantification of intracellular TG levels was performed using a commercial kit (Nanjing Jiancheng Bioengineering Institute, Nanjing, China) according to the manufacturer’s instructions.

The liver tissue was weighed, and absolute ethanol was added according to a 1:9 ratio of weight (g) to volume (ml). The tissues were mechanically homogenized in an ice-water bath, and the homogenate was then centrifuged for 10 min (2,500 r/min). Quantification of the supernatant TG levels was then performed using a commercial kit (Nanjing Jiancheng Bioengineering Institute, Nanjing, China) according to the manufacturer’s instructions.

### Western Blot Analysis

The total protein was extracted from 3T3-L1 adipocytes, liver tissue, and epididymal adipose tissue using radioimmunoprecipitation assay (RIPA) lysis buffer (Solarbio, Beijing, China) containing 1% phenylmethanesulfonyl fluoride (PMSF). The cellular protein concentration was determined using the bicinchoninic acid (BCA) method. The extracted proteins were separated by 10% sodium dodecyl sulfate-polyacrylamide gel electrophoresis (SDS-PAGE). The gels were transferred onto polyvinylidene fluoride (PVDF) membranes (Millipore, Bedford, MA, USA). Each membrane was blocked with 5% bovine serum albumin (BSA) for 30 min and then incubated overnight with primary antibody [AMPK, P-AMPK (Thr172), ACC, P-ACC (Ser79), FAS, HSL, ATGL (1:1,000, Cell Signaling Technology, MA, USA), PPAR-γ, C/EBPα, C/EBPβ (1:200, Santa Cruz Biotechnology, CA, USA), β-actin (1:2,000, ORIGENE, USA)] at 4°C. The next day, the membranes were washed three times with PBS plus Tween 20 (PBST). They were then incubated with goat antirabbit/antimouse secondary antibody conjugated to horseradish peroxidase (1:5,000, R&D Systems, USA) for 1 h at room temperature before they were washed three times with PBST. Subsequently, the protein bands were assessed using an enhanced chemiluminescence kit (Tiangen Biotech CO., LTD., Beijing, China) according to the manufacturer’s instructions. The protein bands were quantified with Image-J and GraphPad Prism 5 (GraphPad Software Inc., San Diego, CA, USA).

### Animal Models and Experimental Protocols

The animal experiments were performed according to international guidelines. The animal experiment protocols were reviewed and approved by the Institutional Animal Ethical Committee of Yunnan Agricultural University. Sixty male 6-week-old C57BL/6J mice (Beijing Vital River Laboratory Animal Technology Co., Ltd, Beijing, China) were kept in a specific-pathogen-free (SPF) facility in 12:12 h day/night cycles in individually ventilated cages (IVCs). The mice were kept 3–4 per cage. The experiments were started on 7-week-old mice. These mice were randomly divided into six groups of 10 mice each: 1) normal diet (ND) group; 2) high-fat diet (HFD) group (with 60% energy from fat; D12492, Research Diets, New Brunswick, NJ, USA); 3) HFD-lovastatin group [with 10 mg/kg body weight (BW) lovastatin] as the positive control; 4) HFD + 0.125 g/kg MOPEE group; 5) HFD + 0.25 g/kg MOPEE group; and 6) HFD + 0.5 g/kg MOPEE group. The MOPEE was dissolved in 0.5% (wt/vol) carboxymethyl-cellulose-Na (CMC-Na). The ND and HFD-only mice were given an equal volume of CMC-Na as the control mice *via* oral gavage. Thus, all mice underwent oral gavage for 14 weeks, and BW was measured every day. Before sacrificing, all mice were fasted for 16 h with free access to water. The mice were then euthanized with CO_2_, and blood was collected *via* the inferior vena cava. Liver and fat tissue were excised, rinsed with PBS, weighed, and stored at −80°C.

### Blood Biochemical Analysis

Blood was collected *via* the inferior vena cava and left at room temperature for 1 h to promote blood coagulation. The blood was centrifuged at 4°C and a centrifugal force of 3,000 × *g* for 10 min to obtain the serum, which was then stored at −80°C. Serum levels of TG, total cholesterol (TC), high-density lipoprotein cholesterol (HDL-C), low-density lipoprotein cholesterol (LDL-C), alanine aminotransferase (ALT), and aspartate aminotransferase (AST) were measured using commercial assay kits (Nanjing Jiancheng Bioengineering Institute, Nanjing, China).

### Epididymal Fat Histopathological Examinations

After freshly isolated epididymal fat tissue was fixed in 10% formalin for 24 h, the tissue was embedded in paraffin and the tissue pieces were cut into 5-μm-thick slices and stained with hematoxylin and eosin (H&E). The tissue structure was observed and photographed under a 200× magnification microscope (Olympus), and the size of the epididymal fat cells was estimated using Image-Pro Plus 6.0 software (Rawak Software Inc., Stuttgart, Germany).

### Statistical Analysis

Values are expressed as mean ± standard error of the mean (SEM). GraphPad Prism5 (GraphPad Software, La Jolla, CA, USA) was used for the statistical analysis. Results were compared by one-way analysis of variance (ANOVA) followed by *post hoc* Tukey-Kramer tests and independent-sample *t* tests. The differences were considered statistically significant at *p* < 0.05.

## Results

### MOPEE Inhibits Adipogenesis in 3T3-L1 Adipocytes

**Figure 1:**
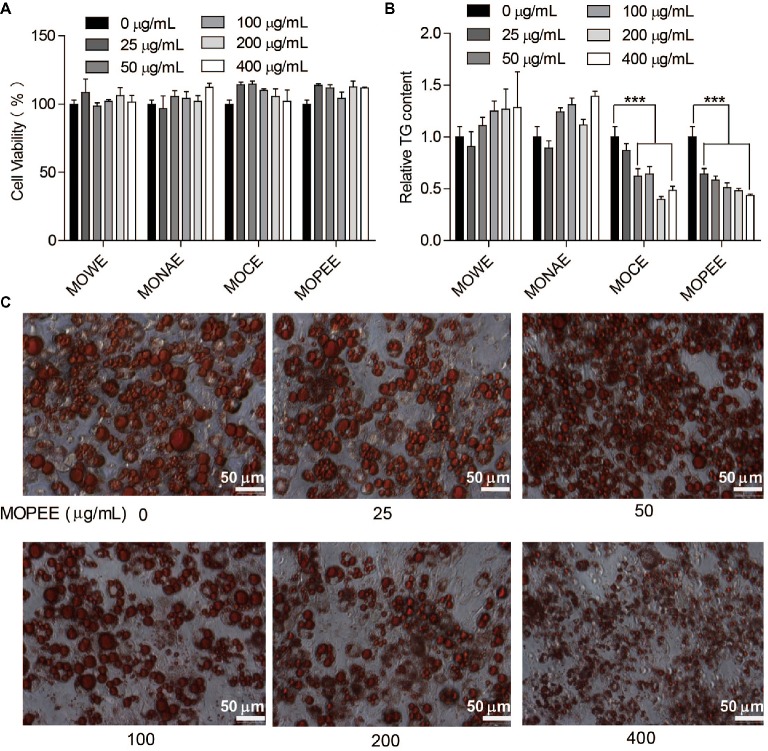
MOPEE inhibits adipogenesis in 3T3-L1 adipocytes. Differentiated 3T3-L1 adipocytes were treated with MOWE, MONAE, MOCE, and MOPEE (0–400 μg/ml) for 24 h, and untreated cells (0 μg/ml) served as the control. **(A)** Cell viability was examined by using a cell counting kit. **(B)** Intracellular triglyceride (TG) content was measured in 3T3-L1 adipocytes. **(C)** Cell differentiation was examined using oil red O staining. Values are expressed as the mean ± SEM (*n* = 3). Significant differences compared to the control (0 μg/ml) are indicated by ***p < 0.001.

To test whether the four extracts of *M. oleifera* leaf had an effect on the survival rate of 3T3-L1 adipocytes, cell viability was measured using a cell counter. We found that the four extracts had no effect on the survival rate of 3T3-L1 adipocytes compared to the untreated control group (Figure 1A). The results showed that the four extracts were not toxic to cells. To detect which extracts could inhibit adipogenesis, 3T3-L1 adipocytes were exposed to the four extracts of *M. oleifera* leaf (25–400 μg/ml) for 24 h, and then the intracellular TG was quantified. The results showed that MOWE and MONAE had no effect on the TG content of 3T3-L1 adipocytes. MOCE (50–400 μg/ml) significantly decreased the TG content in 3T3-L1 adipocytes (*p* < 0.001), but not dose-dependently. MOPEE (25–400 μg/ml) dose-dependently decreased the TG content in 3T3-L1 adipocytes (*p* < 0.001) (Figure 1B). These results indicated that MOPEE has a stronger inhibitory effect on adipogenesis than other extracts. To further clarify the role of MOPEE in inhibiting adipogenesis, we exposed 3T3-L1 adipocytes to MOPEE (25–400 μg/ml) for 24 h and then stained the cells with oil red O dye. The results showed that MOPEE reduced lipid droplet accumulation in the 3T3-L1 adipocytes (Figure 1C). These results indicated that MOPEE inhibited adipogenesis in 3T3-L1 adipocytes.

### MOPEE Inhibits Lipogenesis and Promotes Lipolysis in 3T3-L1 Adipocytes

**Figure 2:**
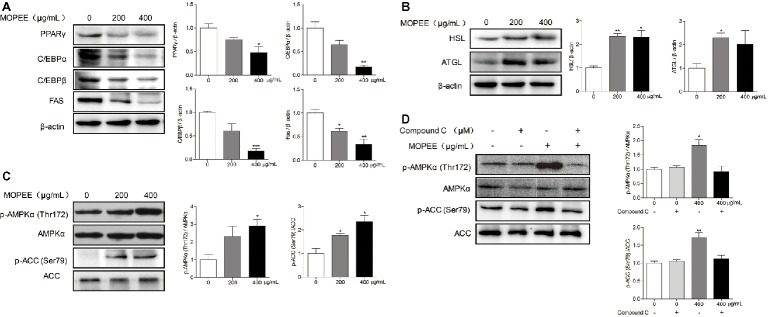
MOPEE inhibits lipogenesis, promotes lipolysis, and activates AMPK signaling in 3T3-L1 adipocytes. Differentiated 3T3-L1 adipocytes were treated with MOPEE (200 and 400 μg/ml) for 24 h, and untreated cells (0 μg/ml) served as the control. **(A)** The levels of lipogenesis-associated proteins (PPARγ, C/EBPα, C/EBPβ, and FAS) and **(B)** lipolysis-associated proteins (ATGL and HSL) were determined by western blot analysis (*n* = 3). Each value was normalized to β-actin and expressed as the mean ± SEM, *n* = 3, *p < 0.05, **p < 0.01, ***p < 0.001. **(C)** The levels of p-AMPK (Thr172), AMPKα, p-ACC (Ser79), and ACC proteins were determined by western blot analysis (*n* = 3). **(D)** 3T3-L1 adipocytes were pretreated with the AMPK inhibitor Compound C (20 μM) for 30 min and then treated with MOPEE (400 μg/ml) for 24 h. The levels of p-AMPK (Thr172), AMPKα, p-ACC (Ser79), and ACC proteins were determined by western blot analysis (*n* = 3). Each value was normalized to AMPKα or ACC and expressed as the mean ± SEM, *n* = 3, *p < 0.05, **p < 0.01, ***p < 0.001.

To determine whether the inhibitory effect of MOPEE on lipogenesis of 3T3-L1 adipocytes was due to downregulation of lipogenesis-associated proteins and upregulation of lipolysis-associated proteins, the expression levels of several proteins were assessed by western blotting. Compared to the differentiated control cells, MOPEE (400 μg/ml) significantly decreased the expression of lipogenesis-associated proteins (PPARγ, C/EBPα, C/EBPβ, and FAS) and significantly increased the expression of the lipolysis-associated protein HSL (Figure 2A,B). These results suggested that MOPEE inhibits adipogenesis by suppressing the expression of lipogenesis-associated proteins and promoting the expression of lipolysis-associated proteins.

### MOPEE Activates AMPK Signaling in 3T3-L1 Adipocytes

To investigate whether the inhibitory effect of MOPEE on lipogenesis of 3T3-L1 adipocytes was due to AMPK activation, we analyzed the expression levels of p-AMPKα and p-ACC by western blotting. We found that MOPEE (400 μg/ml) significantly increased the expression of p-AMPKα and p-ACC in 3T3-L1 adipocytes in a dose-dependent manner (Figure 2C). To determine whether the inhibitory effect of MOPEE on lipogenesis was the result of MOPEE-induced activated AMPK signaling, we used the AMPK inhibitor Compound C to inhibit AMPK activation. As shown in Figure 2D, it was found that after treatment with MOPEE, the protein expression of p-AMPKα (Thr172) and p-ACC (Ser79) was significantly upregulated. However, after treatment with MOPEE and Compound C, the protein expression of p-AMPKα (Thr172) and p-ACC (Ser79) was restored. These results indicated that MOPEE inhibited lipid accumulation in 3T3-L1 adipocytes by activating the AMPK pathway.

### MOPEE Suppresses Lipid Accumulation in HFD Mice

**Figure 3:**
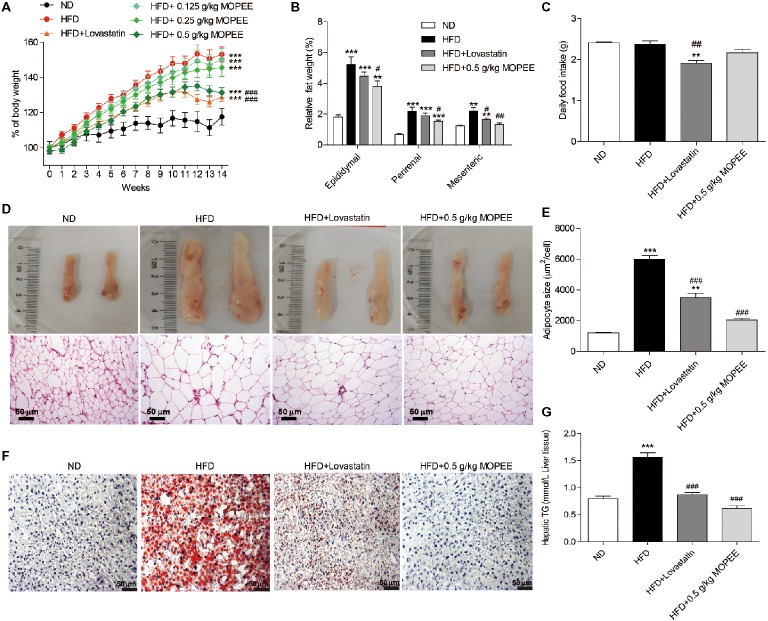
MOPEE suppresses lipid accumulation in high-fat diet (HFD) mice. Changes in the percentage of body weight **(A)**, relative visceral adipocyte tissue weight **(B)**, and daily food intake **(C)** in the ND, HFD, HFD + lovastatin, and HFD + 0.5 g/kg MOPEE group (total *n* = 10/group). **(D)** H&E staining of epididymal fat tissue of the four groups of mice. **(E)** Adipocyte size was monitored in epididymal fat tissue samples by H&E staining. **(F)** Oil red O staining of the liver tissue of the four groups of mice. **(G)** Liver triglyceride (TG) concentration in the four groups of mice. Values are expressed as mean ± SEM, *n* = 10. *p < 0.05, **p < 0.01, ***p < 0.001 versus ND group. ^#^ *p* < 0.05, ^##^ *p* < 0.01, ^###^ *p* < 0.001 versus HFD group.

To test whether MOPEE could prevent HFD-induced obesity, we orally administered MOPEE (0.125, 0.25, and 0.5 g/kg) to HFD mice for 14 weeks. We found that the percentage of BW and relative visceral fat weight were significantly increased in the HFD group (*p* < 0.001) compared to the ND group. However, compared to the HFD group, the percentage of BW of mice in the HFD + 0.5 g/kg MOPEE group was significantly decreased by 21.77% (*p* < 0.001), and the relative weights of epididymal, perirenal, and mesenteric fat tissue were significantly decreased by 1.43% (*p* < 0.05), 0.64% (*p* < 0.05), and 0.87% (*p* < 0.01), respectively (Figure 3A,B). There were no significant differences in food intake (Figure 3C). These results showed that MOPEE inhibits lipid accumulation. Histological analysis of epididymal fat tissue revealed that the mean fat adipocyte size was significantly increased in the HFD group compared to the ND group (*p* < 0.001). However, compared to the HFD group, the mean adipocyte size in the HFD + 0.5 g/kg MOPEE group was significantly decreased (*p* < 0.001) (Figure 3D,E).

Next, we evaluated the inhibitory effect of MOPEE on lipid accumulation in hepatic tissue in HFD mice by oil red O staining and quantitative analysis of hepatic TG. As shown in Figure 3F, lipid droplet accumulation in the HFD group was remarkable compared to that in the ND group. However, lipid droplet accumulation in the HFD + 0.5 g/kg MOPEE group was visibly lower than in the HFD group. The quantitative analysis of lipid content in the hepatic tissue showed that the TG level in the HFD group was significantly increased compared to that in the ND group (*p* < 0.001). However, compared to the HFD mice, the TG levels in the HFD + 0.5 g/kg MOPEE mice were significantly decreased (*p* < 0.001) (Figure 3G). These results suggested that MOPEE reduced hepatic lipid accumulation in HFD mice.

### Effects of MOPEE on Serum TC, TG, HDL-C, LDL-C, AST, and ALT Levels

**Figure 4:**
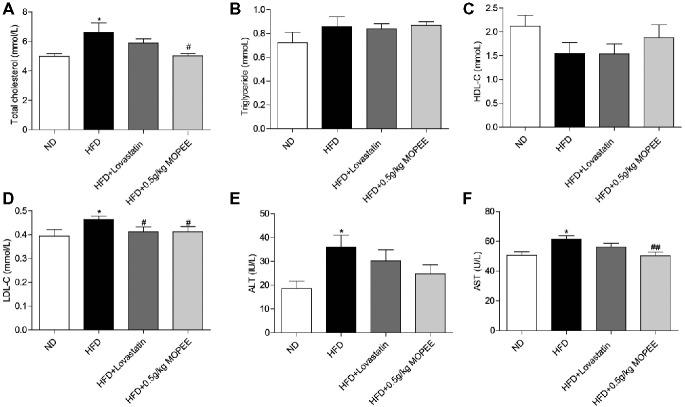
Effects of MOPEE on serum TC, TG, HDL-C, LDL-C, AST, and ALT levels. **(A–D)** Serum TC, TG, HDL-C, and LDL-C levels. (**E, F**) Serum AST and ALT levels. Values are expressed as means ± SEM, *n* = 7. *p < 0.05, **p < 0.01, ***p < 0.001 versus ND group. ^#^ *p* < 0.05, ^##^ *p* < 0.01, ^###^ *p* < 0.001 versus HFD group.

As shown in Figure 4, the serum levels of TC, LDL-C, AST, and ALT were significantly elevated in the HFD group compared to the ND group (*p* < 0.05). However, the serum levels of TC, LDL-C, and AST were significantly decreased in the HFD + 0.5 g/kg MOPEE group compared to the HFD group (*p* < 0.05). In addition, the serum level of TG and HDL-C was nonsignificantly changed in the four groups.

### MOPEE Inhibits Lipogenesis and Promotes Lipolysis in HFD Mice

**Figure 5:**
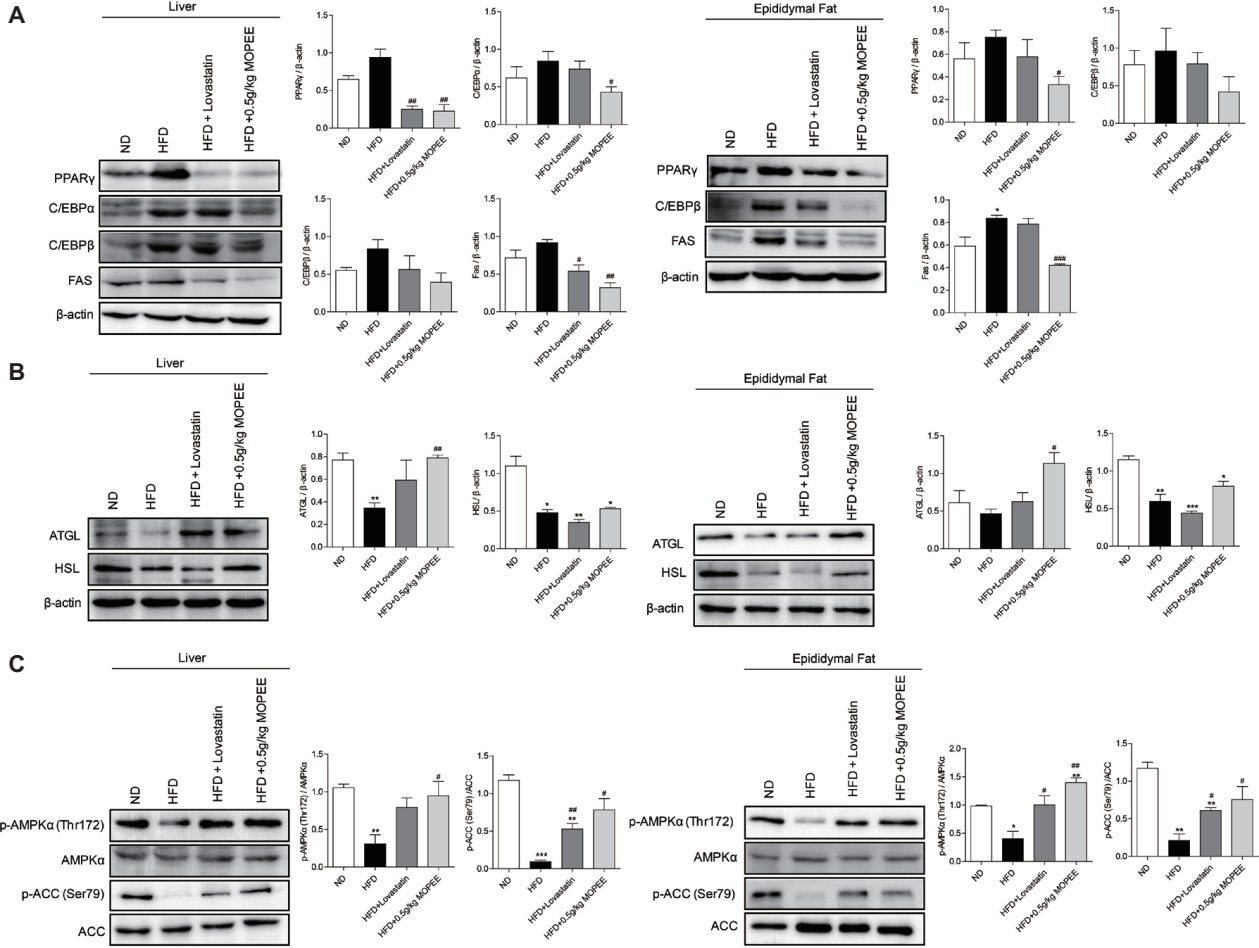
MOPEE inhibits lipogenesis, promotes lipolysis, and activates AMPK signaling in HFD mice. **(A)** The lipogenesis-associated proteins (PPARγ, C/EBPα, C/EBPβ, and FAS) and **(B)** lipolysis-associated proteins (ATGL and HSL) in liver and epididymal fat tissue of mice were determined by western blot analysis. Each value was normalized to β-actin and was expressed as the mean ± SEM, *n* = 3, *p < 0.05, **p < 0.01, ***p < 0.001 versus ND group. ^#^ *p* < 0.05, ^##^ *p* < 0.01, ^###^ *p* < 0.001 versus HFD group. **(C)** The p-AMPK (Thr172), AMPKα, p-ACC (Ser79), and ACC proteins in liver and epididymal fat tissue of mice were determined by western blot analysis. Each value was normalized to AMPKα or ACC and expressed as the mean ± SEM, *n* = 3, *p < 0.05, **p < 0.01, ***p < 0.001 versus ND group. ^#^ *p* < 0.05, ^##^ *p* < 0.01, ^###^ *p* < 0.001 versus HFD group.

To investigate whether lipid accumulation inhibition by MOPEE in HFD mice was associated with inhibition of fat synthesis and promotion of lipolysis, the expression levels of lipogenesis-associated proteins and lipolysis-associated proteins were analyzed by western blotting. As shown in Figure 5A,B, compared to the ND group, the expression of the lipogenesis-associated protein FAS in the adipose tissue in the HFD group was significantly increased, and the lipolysis-associated protein HSL in the hepatic and epididymal fat tissue in the HFD group was significantly decreased (*p* < 0.05). However, compared to the HFD group, the expression of the adipogenesis-associated proteins PPARγ and FAS in the hepatic and epididymal fat tissue in the HFD + 0.5 g/kg MOPEE group was significantly decreased, and the expression of the lipolysis-associated protein ATGL was significantly increased (*p* < 0.05).

### MOPEE Activates AMPK Signaling in HFD Mice

To determine whether the inhibitory effect of MOPEE on lipid accumulation in HFD mice was due to AMPK activation, we analyzed the expression of p-AMPKα and p-ACC in both liver and epididymal fat tissue. We found that the protein levels of p-AMPKα (Thr172) and p-ACC (Ser79) were significantly decreased in both liver and epididymal fat tissue in the HFD group compared to the ND group (*p* < 0.05). However, compared to HFD mice, MOPEE markedly promoted the protein expression levels of p-AMPKα (Thr172) and p-ACC (Ser79) in liver and epididymal fat tissue in HFD mice (*p* < 0.05) (Figure 5C). These results suggested that MOPEE decreased the adiposity through activation of the AMPK pathway.

### UPLC-QTOF-MS/MS Analysis of MOPEE

**Figure 6:**
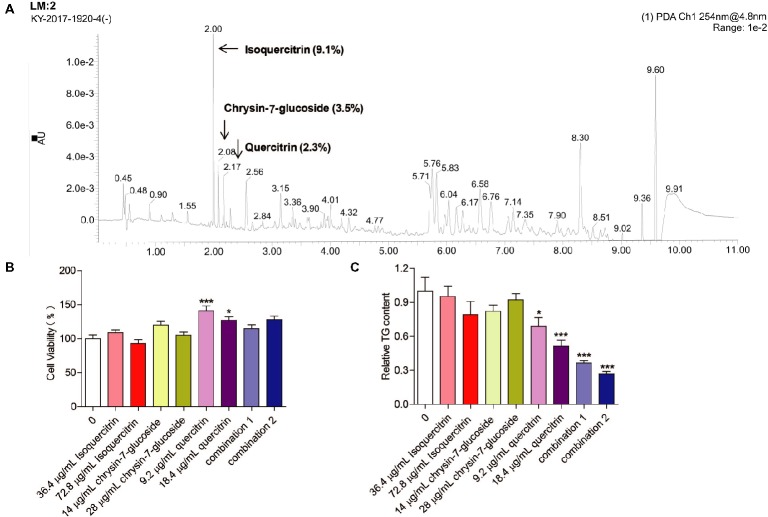
Effect of relatively high-content compounds in MOPEE on the TG level in 3T3-L1 adipocytes. **(A)** UPLC-QTOF-MS/MS chromatogram of MOPEE. 3T3-L1 adipocytes were treated with isoquercitrin (36.4 and 72.8 μg/ml), chrysin-7-glucoside (14 and 28 μg/ml), quercitrin (9.2 and 18.4 μg/ml), combination 1 (36.4 μg/ml isoquercitrin + 14 μg/ml glucoside + 9.2 μg/ml quercitrin), and combination 2 (72.8 μg/ml isoquercitrin + 28 μg/ml glucoside + 18.4 μg/ml quercitrin) for 24 h, and untreated cells (0 μg/ml) served as the control. **(B)** Cell viability was examined by MTT. **(C)** Intracellular TG content was measured in 3T3-L1 adipocytes. Values are expressed as the mean ± SEM (*n* = 3). Significant differences compared to the control (0 μg/ml) are indicated by *p < 0.05, ***p < 0.001.

Three compounds (isoquercitrin, chrysin-7-glucoside, and quercitrin), which are known constituents extracted from *M. oleifera* leaves, were analyzed by UPLC-QTOF-MS/MS. The retention times of the three compounds were 2.00, 2.08, and 2.17 min, respectively. The relative quantities were 9.1, 3.5, and 2.3%, respectively (Figure 6A). Chemically, quercitrin, isoquercitrin and chrysin-7-glucoside are flavonoid glycosides.

### Quercitrin and Combinations of Isoquercitrin, Chrysin-7-Glucoside, and Quercitrin Inhibit Adipogenesis in 3T3-L1 Adipocytes

To test whether isoquercitrin, crysin-7-glucoside, quercitrin, and combinations of the three compounds have an effect on the survival rate of 3T3-L1 adipocytes, cell viability was measured by MTT. We found that isoquercitrin, chrysin-7-glucoside, and the combinations of the three compounds had no effect on the survival rate of 3T3-L1 adipocytes compared to the untreated control group (Figure 6B). Quercitrin significantly increased the cell survival rate (*p* < 0.05) (Figure 6B). To detect which compounds or combinations could inhibit adipogenesis, 3T3-L1 adipocytes were exposed to isoquercitrin, chrysin-7-glucoside, quercitrin, and the two combinations for 24 h, and intracellular TG was then quantified. The results showed that isoquercitrin and chrysin-7-glucoside had no effect on the TG content of 3T3-L1 adipocytes. Quercitrin and the two combinations of the three compounds significantly decreased the TG content in 3T3-L1 adipocytes in a dose-dependent manner (*p* < 0.05) (Figure 6C). These results indicated that quercitrin is the main component of MOPEE to inhibit adipogenesis. In addition, isoquercitrin, crysin-7-glucoside, and quercitrin have synergistic effects regarding adipogenesis inhibition.

## Discussion

The tropical tree *M. oleifera* is a well-known source of traditional medicines. Recent studies have also reported that *M. oleifera* leaf extracts protect against obesity in animal models. For instance, *M. oleifera* leaf aqueous ethanol extract prevented weight gain in diet-induced obese mice (Attakpa et al., 2017). Fermented *M. oleifera* methanol extract decreased the hepatic lipid accumulation in HFD mice, and genes involved in liver lipid metabolism were upregulated (Joung et al., 2017). Isothiocyanate-rich *M. oleifera* extract reduced weight gain and hepatic adiposity while increasing lipolysis (Waterman et al., 2015). In addition, many studies reported that *M. oleifera* leaf extract has antihyperlipidemic properties in animal models (Adisakwattana and Chanathong, 2011; Toma et al., 2015). *M. oleifera* leaf methanol extract significantly reduced the serum levels of TG, TC, and LDL-C and increased the serum level of HDL-C in diabetic rats (Olayaki et al., 2015). *M. oleifera* leaf aqueous ethanol extract significantly decreased the levels of cholesterol and TG in fructose-induced hypertensive rats compared to control preparations (Geleta et al., 2016). After 12 weeks of feeding rabbits with high-cholesterol diets, animals given *M. oleifera* leaf aqueous extracts had significantly lower cholesterol levels (Chumark et al., 2008). Similar aqueous extracts of *M. oleifera* leaves significantly reduced TC levels in the serum (14.4%), liver (6.4%), and kidneys (11.1%) in HFD rats (Ghasi et al., 2000). The above studies indicate that *M. oleifera* leaf aqueous extract, hydroalcohol extract, and alcohol (methanol or ethanol) extract are the main *M. oleifera* leaf extracts that have been used to study the antiobesity and antihyperlipidemic effects of *M. oleifera*. In this study, we used MOPEE for the first time and demonstrated its role and mechanism in inhibiting lipid accumulation.

Three bioactive phytochemicals (quercitrin, chlorogenic acid, and benzylamine) from *M. oleifera* leaves have previously been evaluated for therapeutic efficacy against dyslipidemia. Studies have shown that quercitrin has antidyslipidemic and antiatherosclerosis effects in high-carbohydrate diet (HCD) and HFD rabbits (Juźwiak et al., 2005; Mbikay, 2012) and antidyslipidemic, hypotensive, and antidiabetic effects in obese Zucker rats (Rivera et al., 2008). In addition, chlorogenic acid reduced plasma TC and TG in obese Zucker rats and HFD mice (Cho et al., 2010) and reduced blood lipids in streptozotocin-induced diabetic rats (Karthikesan et al., 2010). In insulin-resistant C57BL/6 mice, alkaloid-benzylamine decreased weight gain, fasting plasma glucose, and TC and increased glucose tolerance (Iffiú-Soltész et al., 2010). We found that the compounds in MOPEE with relatively high levels are isoquercitrin (9.1%), chrysin-7-glucoside (3.5%), and quercitrin (2.3%). We also found that quercitrin is the main component of MOPEE to inhibit adipogenesis. In addition, isoquercitrin, crysin-7-glucoside, and quercitrin have synergistic effects in inhibiting adipogenesis.

Based on a large number of *in vivo* studies, various extracts of *M. oleifera* leaves appear to be safe and nontoxic in various animals at the commonly used doses (Stohs and Hartman, 2015). For example, rats had no adverse effects after intragastric administration of 2,000 mg/kg *M. oleifera* leaf water extract (Asiedu-Gyekye et al., 2014). In addition, a study found that the median lethal dose (LD50) of *M. oleifera* leaf ethanol extract in mice was 6.4 g/kg (Bakre et al., 2013). *M. oleifera* leaf methanol extract (50, 100, 200, and 400 mg/kg) promoted weight gain in rats in a dose-dependent manner (Oyagbemi et al., 2013). In our study, a 500 mg/kg dose of MOPEE had no effect on visceral weight and serum ALT and AST levels in HFD mice, demonstrating the safety of this dose.

Fat accumulation is considered to be a critical step in the development of obesity; controlling the process would allow effective control of the progress of obesity (Rosen and Spiegelman, 2006). Fat accumulation involves the proliferation of adipocytes in adipose tissue and the differentiation of preadipocytes. The volume and degree of differentiation of single adipocytes are closely related to intracellular TG accumulation quantity. The differentiation process of adipocytes is regulated by a complex network of transcription factors. The process mainly involves two types of transcription factors: PPARγ and C/EBPs. In the early stage of differentiation, the transcription factor C/EBPβ is activated first, and in the later stage, PPARγ and C/EBPα are activated one after another. PPARγ and C/EBPα synergistically regulate downstream differentiation genes and promote fat synthesis (Feige and Auwerx, 2007). Therefore, specific regulation of the expression of the transcription factors C/EBPβ, C/EBPα, and PPARγ has become a target of obesity treatment (Rayalam et al., 2008). In this study, MOPEE significantly decreased the protein expression levels of C/EBPα, C/EBPβ, and PPARγ in adipocytes, and MOPEE significantly decreased the protein expression levels of PPARγ in HFD mice liver and epididymal fat tissue, indicating that MOPEE inhibits adipocyte differentiation. FAS is a key enzyme in the fatty acid synthesis pathway and plays an important role in fat synthesis (Wakil and Abuelheiga, 2009). Research has shown that pomegranate extract reduces BW by inhibiting the FAS activity (Wu et al., 2013). The results of this study indicate that MOPEE significantly reduces the expression of FAS protein in adipocytes and liver and epididymal fat tissue and then significantly reduces intracellular TG production and fat accumulation. HSL and ATGL are two rate-limiting enzymes of intracellular TG hydrolysis. In this study, MOPEE significantly promoted the protein expression of HSL and ATGL in adipocytes, and MOPEE significantly promoted the protein expression of ATGL in HFD mice liver and epididymal fat tissue, indicating that MOPEE enhances lipolysis activity.

AMPK is a key regulator of cellular glucose and lipid metabolism, and its activation enhances insulin sensitivity in various tissues, promotes energy generation (glucose transport and fatty acid oxidation), inhibits energy expenditure (lipid synthesis, protein synthesis, and gluconeogenesis), inhibits the release of fatty acids, and promotes the fatty acid degradation in adipocytes, thus having the potential therapeutic effects on metabolic syndrome, diabetes, and so on (Daval et al., 2006). AMPK is a member of the serine/threonine protein kinase family. Under the action of upstream signaling molecules, phosphorylation of AMPK occurs at the Thr172 site and it becomes activated. Activated AMPK inhibits adipocyte differentiation by inhibiting the key molecules in adipocyte differentiation. Research has shown that the AMPK activator A-769662 can downregulate the expression of PPARγ, C/EBPα, and FAS, thereby inhibiting the adipocyte differentiation (Zhou et al., 2009). ACC is a downstream molecule of AMPK that regulates the synthesis of fatty acids. Phosphorylation of AMPK promotes phosphorylation of ACC, resulting in inactivation of ACC, thereby reducing the free fatty acid production. In this study, MOPEE significantly promoted the protein expression levels of p-AMPKα (Thr172) and p-ACC (Ser79) in 3T3-L1 adipocytes and HFD mice liver and epididymal fat tissues. These results indicate that MOPEE can reduce lipid deposition in liver and adipose tissue and inhibit 3T3-L1 adipogenic differentiation, which may be related to the upregulation of p-AMPK protein expression.

Based on these findings, we conclude that MOPEE inhibits fat accumulation by inhibiting the adipogenesis and promoting the lipolysis, and this process is related to AMPK activation.

## Author Contributions

JS and YT conceived and designed the experiments. JX, YW, WW, XF, TY, LP, SS, LF, LT, CY, RS, and RX performed the experiments. JX, YW, and WW analyzed the data. JS, YT, and JX wrote the manuscript. All authors reviewed the manuscript.

### Conflict of Interest Statement

The authors declare that the research was conducted in the absence of any commercial or financial relationships that could be construed as a potential conflict of interest.

## References

[ref1] Abdull Razis A. F. Ibrahim M. D. Kntayya S. B. (2014). Health benefits of *Moringa oleifera*. Asian Pac. J. Cancer Prev. 15, 8571–8576. 10.7314/APJCP.2014.15.20.8571, PMID: 25374169

[ref2] Adisakwattana S. Chanathong B. (2011). Alpha-glucosidase inhibitory activity and lipid-lowering mechanisms of *Moringa oleifera* leaf extract. Eur. Rev. Med. Pharmacol. Sci. 15, 803–808. , PMID: 21780550

[ref3] Asiedu-Gyekye I. J. Frimpong-Manso S. Awortwe C. Antwi D. A. Nyarko A. K. (2014). Micro- and macroelemental composition and safety evaluation of the nutraceutical *Moringa oleifera* leaves. J. Toxicol. 2014:786979. 10.1155/2014/786979, PMID: 25136361 PMC4129914

[ref4] Attakpa E. S. Sangaré M. M. Béhanzin G. J. Ategbo J. M. Seri B. Khan N. A. (2017). *Moringa olifeira* Lam. stimulates activation of the insulin-dependent akt pathway. Antidiabetic effect in a diet-induced obesity (DIO) mouse model. Folia Biol. 63, 42–51, PMID: 28557705 10.14712/fb2017063020042

[ref5] Bakre A. G. Aderibigbe A. O. Ademowo O. G. (2013). Studies on neuropharmacological profile of ethanol extract of *Moringa oleifera* leaves in mice. J. Ethnopharmacol. 149, 783–789. 10.1016/j.jep.2013.08.006, PMID: 23933316

[ref6] Bullon P. Marinaguilar F. Romanmalo L. (2016). AMPK/mitochondria in metabolic diseases. Exp. Suppl. 107, 129–152. 10.1007/978-3-319-43589-3_6, PMID: 27812979

[ref7] Cho A. S. Jeon S. M. Kim M. J. Yeo J. Seo K. I. Choi M. S. (2010). Chlorogenic acid exhibits anti-obesity property and improves lipid metabolism in highfat diet-induced-obese mice. Food Chem. Toxicol. 48, 937–943. 10.1016/j.fct.2010.01.003, PMID: 20064576

[ref8] Chumark P. Khunawat P. Sanvarinda Y. Phornchirasilp S. Morales N. P. Phivthong-Ngam L. (2008). The in vitro and ex vivo antioxidant properties, hypolipidaemic and antiatherosclerotic activities of water extract of *Moringa oleifera* Lam. leaves. J. Ethnopharmacol. 116, 439–446. 10.1016/j.jep.2007.12.010, PMID: 18249514

[ref9] Daval M. Foufelle F. Ferré P. (2006). Functions of AMP-activated protein kinase in adipose tissue. J. Physiol. 574, 55–62. 10.1113/jphysiol.2006.111484, PMID: 16709632 PMC1817807

[ref10] Feige J. N. Auwerx J. (2007). Transcriptional coregulators in the control of energy homeostasis. Trends Cell Biol. 17, 292–301. 10.1016/j.tcb.2007.04.001, PMID: 17475497

[ref11] Figarola J. L. Rahbar S. (2013). Small-molecule COH-SR4 inhibits adipocyte differentiation via AMPK activation. Int. J. Mol. Med. 31, 1166–1176. 10.3892/ijmm.2013.1313, PMID: 23525347

[ref12] Geleta B. Makonnen E. Debella A. Tadele A. (2016). In vivo antihypertensive and antihyperlipidemic effects of the crude extracts and fractions of moringa stenopetala (Baker f.) cufod. leaves in rats. Front. Pharmacol. 7:97. 10.3389/fphar.2016.00097, PMID: 27148056 PMC4838630

[ref13] Ghasi S. Nwobodo E. Ofili J. O. (2000). Hypocholesterolemic effects of crude extract of leaf of *Moringa oleifera* Lam in high-fat diet fed wistar rats. J. Ethnopharmacol. 69, 21–25. 10.1016/S0378-8741(99)00106-3, PMID: 10661880

[ref14] Helmy S. A. Nfs M. Elaby S. M. Maa G. (2017). Hypolipidemic effect of *Moringa oleifera* Lam leaf powder and its extract in diet-induced hypercholesterolemic rats. J. Med. Food 20, 755–762. 10.1089/jmf.2016.0155, PMID: 28459609

[ref15] Iffiú-Soltész Z. Wanecq E. Lomba A. Portillo M. P. Pellati F. Szöko E. (2010). Chronic benzylamine administration in the drinking water improves glucose tolerance, reduces body weight gain and circulating cholesterol in high-fat diet-fed mice. Pharmacol. Res. 61, 355–363. 10.1016/j.phrs.2009.12.014, PMID: 20045461

[ref16] Joung H. Kim B. Park H. Lee K. Kim H. H. Sim H. C. (2017). Fermented *Moringa oleifera* decreases hepatic adiposity and ameliorates glucose intolerance in high-fat diet-induced obese mice. J. Med. Food 20, 439–447. 10.1089/jmf.2016.3860, PMID: 28504910

[ref17] Juźwiak S. Wójcicki J. Mokrzycki K. Marchlewicz M. Białecka M. Wenda-Rózewicka L. (2005). Effect experimental hyperlipidemia and atherosclerosis in rabbits. Pharmacol. Rep. 57, 604–609, PMID: 16227643

[ref18] Karthikesan K. Pari L. Menon V. P. (2010). Combined treatment of tetrahydrocurcumin and chlorogenic acid exerts potential antihyperglycemic effect on streptozotocin-nicotinamideinduced diabetic rats. Gen. Physiol. Biophys. 29, 23–30. 10.4149/gpb_2010_01_23, PMID: 20371877

[ref19] Lai C. S. Liao S. N. Tsai M. L. Kalyanam N. Majeed M. Majeed A. (2015). Calebin-A inhibits adipogenesis and hepatic steatosis in high-fat diet-induced obesity via activation of AMPK signaling. Mol. Nutr. Food Res. 59, 1883–1895. 10.1002/mnfr.201400809, PMID: 26108684

[ref20] Lee Y. H. Kim Y. S. Song M. Lee M. Park J. Kim H. (2015). A herbal formula HT048, citrus unshiu and crataegus pinnatifida, prevents obesity by inhibiting adipogenesis and lipogenesis in 3T3-L1 preadipocytes and HFD-induced obese rats. Molecules 20, 9656–9670. 10.3390/molecules20069656, PMID: 26016552 PMC6272291

[ref21] Liou C. J. Wu S. J. Chen L. C. Yeh K. W. Chen C. Y. Huang W. C. (2017). Acacetin from traditionally used *Saussurea involucrata* Kar. et Kir. suppressed adipogenesis in 3T3-L1 adipocytes and attenuated lipid accumulation in obese mice. Front. Pharmacol. 8:589. 10.3389/fphar.2017.00589, PMID: 28900399 PMC5581916

[ref22] Martin K. Mani M. Mani A. (2015). New targets to treat obesity and metabolic syndrome. Eur. J. Pharmacol. 763, 64–74. 10.1016/j.ejphar.2015.03.093, PMID: 26001373 PMC4573317

[ref23] Mbikay M. (2012). Therapeutic potential of *Moringa oleifera* leaves in chronic hyperglycemia and dyslipidemia: a review. Front. Pharmacol. 3:24. 10.3389/fphar.2012.00024, PMID: 22403543 PMC3290775

[ref24] Mottillo E. P. Desjardins E. M. Fritzen A. M. Zou V. Z. Crane J. D. Yabut J. M. (2017). FGF21 does not require adipocyte AMP-activated protein kinase (AMPK) or the phosphorylation of acetyl-CoA carboxylase (ACC) to mediate improvements in whole-body glucose homeostasis. Mol. Metab. 6, 471–481. 10.1016/j.molmet.2017.04.001, PMID: 28580278 PMC5444097

[ref25] Ndong M. Uehara M. Katsumata S. Sato S. Suzuki K. (2007). Preventive effects of *Moringa oleifera* (Lam) on hyperlipidemia and hepatocyte ultrastructural changes in iron deficient rats. Biosci. Biotechnol. Biochem. 71, 1826–1833. 10.1271/bbb.60644, PMID: 17690476

[ref26] Nielsen T. S. Jessen N. Jørgensen J. O. Møller N. Lund S. (2014). Dissecting adipose tissue lipolysis: molecular regulation and implications for metabolic disease. J. Mol. Endocrinol. 52, R199–R222. 10.1530/JME-13-0277, PMID: 24577718

[ref27] O’neill S. O’driscoll L. (2015). Metabolic syndrome: a closer look at the growing epidemic and its associated pathologies. Obes. Rev. 16, 1–12. 10.1111/obr.12229, PMID: 25407540

[ref28] Olayaki L. A. Irekpita J. E. Yakubu M. T. Ojo O. O. (2015). Methanolic extract of *Moringa oleifera* leaves improves glucose tolerance, glycogen synthesis and lipid metabolism in alloxan-induced diabetic rats. J. Basic Clin. Physiol. Pharmacol. 26, 585–593. 10.1515/jbcpp-2014-0129, PMID: 26124050

[ref29] Oyagbemi A. A. Omobowale T. O. Azeez I. O. Abiola J. O. Adedokun R. A. Nottidge H. O. (2013). Toxicological evaluations of methanolic extract of *Moringa oleifera* leaves in liver and kidney of male Wistar rats. J. Basic Clin. Physiol. Pharmacol. 24, 307–312. 10.1515/jbcpp-2012-0061, PMID: 23509212

[ref30] Rayalam S. Dellafera M. A. Baile C. A. (2008). Phytochemicals and regulation of the adipocyte life cycle. J. Nutr. Biochem. 19, 717–726. 10.1016/j.jnutbio.2007.12.007, PMID: 18495457

[ref31] Rivera L. Morón R. Sánchez M. Zarzuelo A. Galisteo M. (2008). Quercetin ameliorates metabolic syndrome and improves the inflammatory status in obese Zucker rats. Obesity 16, 2081–2087. 10.1038/oby.2008.315, PMID: 18551111

[ref32] Rosen E. D. Spiegelman B. M. (2006). Adipocytes as regulators of energy balance and glucose homeostasis. Nature 444, 847–853. 10.1038/nature05483, PMID: 17167472 PMC3212857

[ref33] Sangkitikomol W. Rocejanasaroj A. Tencomnao T. (2014). Effect of Moringa oleifera on advanced glycation end-product formation and lipid metabolism gene expression in HepG2 cells. Genet. Mol. Res. 13, 723–735. 10.4238/2014.January.29.3, PMID: 24615037

[ref34] Sharma B. R. Oh J. Kim H. A. Kim Y. J. Jeong K. S. Rhyu D. Y. (2015). Anti-obesity effects of the mixture of *Eriobotrya japonica* and *Nelumbo nucifera* in adipocytes and high-fat diet-induced obese mice. Am. J. Chin. Med. 43, 681–694. 10.1142/S0192415X15500421, PMID: 26133751

[ref35] Stohs S. J. Hartman M. J. (2015). Review of the safety and efficacy of *Moringa oleifera*. Phytother. Res. 29, 796–804. 10.1002/ptr.5325, PMID: 25808883 PMC6680322

[ref36] Toma A. Makonnen E. Mekonnen Y. Debella A. Adisakwattana S. (2015). Antidiabetic activities of aqueous ethanol and n-butanol fraction of *Moringa stenopetala* leaves in streptozotocin-induced diabetic rats. BMC Complement. Altern. Med. 15, 242–249. 10.1186/s12906-015-0779-0 26187590 PMC4506633

[ref37] Wakil S. J. Abuelheiga L. A. (2009). Fatty acid metabolism: target for metabolic syndrome. J. Lipid Res. 50, S138–S143. 10.1194/jlr.R800079-JLR200, PMID: 19047759 PMC2674721

[ref38] Waterman C. Rojas-Silva P. Tumer T. B. Kuhn P. Richard A. J. Wicks S. (2015). Isothiocyanate-rich *Moringa oleifera* extract reduces weight gain, insulin resistance and hepatic gluconeogenesis in mice. Mol. Nutr. Food Res. 59, 1013–1024. 10.1002/mnfr.201400679, PMID: 25620073 PMC4456298

[ref39] Wu D. Ma X. Tian W. (2013). Pomegranate husk extract, punicalagin and ellagic acid inhibit fatty acid synthase and adipogenesis of 3T3-L1 adipocyte. J. Funct. Foods. 5, 633–641. 10.1016/j.jff.2013.01.005

[ref40] Yassa H. D. Tohamy A. F. (2014). Extract of *Moringa oleifera* leaves ameliorates streptozotocin-induced diabetes mellitus in adult rats. Acta Histochem. 116, 844–854. 10.1016/j.acthis.2014.02.002, PMID: 24657072

[ref41] Zhou Y. Wang D. Zhu Q. Gao X. Yang S. Xu A. (2009). Inhibitory effects of A-769662, a novel activator of AMP-activated protein kinase, on 3T3-L1 adipogenesis. Biol. Pharm. Bull. 32, 993–998. 10.1248/bpb.32.993, PMID: 19483304

